# Effect of omission of surgery on survival in patients aged 80 years and older with early‐stage hormone receptor‐positive breast cancer

**DOI:** 10.1002/bjs.11568

**Published:** 2020-04-07

**Authors:** A. Z. de Boer, N. A. de Glas, P. J. Marang‐van de Mheen, O. M. Dekkers, S. Siesling, L. de Munck, K. M. de Ligt, G. J. Liefers, J. E. A. Portielje, E. Bastiaannet

**Affiliations:** ^1^ Department of Surgery Leiden the Netherlands; ^2^ Department of Medical Oncology Leiden the Netherlands; ^3^ Department of Medical Decision‐Making Leiden the Netherlands; ^4^ Department of Clinical Epidemiology Leiden University Medical Centre Leiden the Netherlands; ^5^ Department of Research and Development, Netherlands Comprehensive Cancer Organization Utrecht the Netherlands; ^6^ Department of Health Technology and Services Research, Technical Medical Centre University of Twente Enschede the Netherlands

## Abstract

**Background:**

Surgery is increasingly being omitted in older patients with operable breast cancer in the Netherlands. Although omission of surgery can be considered in frail older patients, it may lead to inferior outcomes in non‐frail patients. Therefore, the aim of this study was to evaluate the effect of omission of surgery on relative and overall survival in older patients with operable breast cancer.

**Methods:**

Patients aged 80 years or older diagnosed with stage I–II hormone receptor‐positive breast cancer between 2003 and 2009 were selected from the Netherlands Cancer Registry. An instrumental variable approach was applied to minimize confounding, using hospital variation in rate of primary surgery. Relative and overall survival was compared between patients treated in hospitals with different rates of surgery.

**Results:**

Overall, 6464 patients were included. Relative survival was lower for patients treated in hospitals with lower compared with higher surgical rates (90·2 *versus* 92·4 per cent respectively after 5 years; 71·6 *versus* 88·2 per cent after 10 years). The relative excess risk for patients treated in hospitals with lower surgical rates was 2·00 (95 per cent c.i. 1·17 to 3·40). Overall survival rates were also lower among patients treated in hospitals with lower compared with higher surgical rates (48·3 *versus* 51·3 per cent after 5 years; 15·0 *versus* 19·7 per cent after 10 years respectively; adjusted hazard ratio 1·07, 95 per cent c.i. 1·00 to 1·14).

**Conclusion:**

Omission of surgery is associated with worse relative and overall survival in patients aged 80 years or more with stage I–II hormone receptor‐positive breast cancer. Future research should focus on the effect on quality of life and physical functioning.

## Graphical abstract

Although the selection criteria for omission of surgery in early‐stage breast cancer are ill defined in guidelines, the percentage of patients receiving surgical treatment declines in older age groups. This study evaluated the effect of omission of surgery on survival in patients aged 80 years and older. Omission of surgery was associated with worse relative and overall survival, but no effect was observed in the first 5 years.

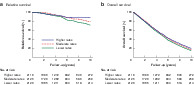

Surgery superior

## Abstract (es)

**Antecedentes:**

En los Países Bajos cada vez es más frecuente descartar la cirugía en pacientes mayores con cáncer de mama operable. Aunque la omisión de la cirugía puede ser adecuada en pacientes mayores frágiles, ello puede determinar peores resultados en pacientes no frágiles. Por tanto, el objetivo de este estudio fue evaluar el efecto de omitir la cirugía en la supervivencia relativa y en la supervivencia global en pacientes mayores con cáncer de mama operable.

**Métodos:**

A partir del Registro de Cáncer de los Países Bajos se seleccionaron las pacientes de ≥ 80 años de edad diagnosticadas de cáncer de mama entre 2003‐2009 en estadios 1‐2 y con receptores hormonales positivos. Se aplicó un método de variables instrumentales para minimizar los factores de confusión utilizando la tasa de variación hospitalaria de la cirugía primaria. Se compararon las supervivencias relativa y global de las pacientes tratadas en hospitales con diferentes tasas de cirugía.

**Resultados:**

Se incluyeron 6.464 pacientes. La supervivencia relativa fue menor en las pacientes tratadas en hospitales con tasas quirúrgicas más bajas en comparación con las tratadas en hospitales con tasas altas (90,2% *versus* 92,4% a los 5 años y 71,6% *versus* 88,2% a los 10 años, respectivamente). El exceso de riesgo relativo para las pacientes tratadas en hospitales con tasas quirúrgicas más bajas fue de 2,00 (i.c. del 95% 1,17‐3,40). La supervivencia global también fue menor para las pacientes tratadas en hospitales con tasas quirúrgicas más bajas en comparación con las más altas (48,3% *versus* 51,3% a los 5 años y 15,0% *versus* 19,7% a los 10 años, respectivamente, cociente de riesgos instantáneos, *hazard ratio*, HR, ajustado 1,07) i.c. del 95% 1,00‐1,14)).

**Conclusión:**

Omitir la cirugía se asocia con una peor supervivencia relativa y global en pacientes de ≥ 80 años con cáncer de mama en estadios 1‐2 y receptores hormonales positivos. Las investigaciones futuras deberían centrarse en el efecto de este enfoque en la calidad de vida y la funcionalidad física.

## Introduction

The number of older patients with breast cancer is increasing owing to ageing of Western populations^1^, ^2^. This age group differs in terms of co‐morbidity, physical and cognitive functioning, and demands a personalized approach to cancer treatment. Less extensive treatments are often given when co‐morbidity or a limited life expectancy is assumed to interfere with treatment benefit. Selection criteria for treatments are, however, poorly defined in guidelines as evidence from RCTs is lacking^3^. Consequently, treatment variation is seen across countries, regions and hospitals^4^, ^5^, ^6^.

Previous studies^7^, ^8^, ^9^ have shown that the percentage of older patients who do not undergo primary surgical treatment has increased over the past decade in the Netherlands. Most of these patients receive primary endocrine therapy instead of surgery. The assumption is that, with primary endocrine therapy, disruption of daily life may be minimized and risks of surgery can be avoided. After an uncertain length of time, disease progression will, however, occur and a change of treatment is required. Endocrine therapy can also have many side‐effects affecting quality of life, especially in older patients^10^, ^11^.

International recommendations^3^ state that primary endocrine therapy should be considered only in patients with a life expectancy of 2–3 years and who are unfit for, or refuse, surgery. Although RCTs comparing surgical treatment and tamoxifen monotherapy reported high rates of local progression in patients treated with tamoxifen alone, none showed a survival difference before 3 years^12^, ^13^. The applicability of data from these studies, undertaken in the 1980s, to current practice is questionable. Hormone receptor testing is now mandatory, and aromatase inhibitors have been shown to be superior to tamoxifen in both (neo)adjuvant and metastatic settings^14^, ^15^, ^16^. Furthermore, multiple lines of endocrine agents are available^13^, ^17^, ^18^. In addition, advances in anaesthetic techniques have made breast surgery a safe procedure^17^, even in the very old^19^. Moreover, previous RCTs included only older patients who were considered fit enough to undergo surgery, which limits the generalizability of the results to the general population of older patients with breast cancer^12^.

Population‐based data may provide more insight into the effect of omission of surgery in the older patient population in current practice. Comparison of patients treated with and without surgery in observational data is, however, susceptible to confounding by indication. Although statistical techniques may adjust for measured confounders, such as age and co‐morbidity, residual confounding by unmeasured factors related to frailty is likely to be present. The variation in omission of surgery among hospitals provides the opportunity to use the instrumental variable approach, an alternative method to minimize confounding. The aim of this study was to evaluate the effect of omission of surgery on relative and overall survival by comparing the outcomes of patients treated in hospitals with different rates of primary surgery.

## Methods

Patients aged 80 years or older diagnosed with stage I–II hormone receptor‐positive breast cancer between 2003 and 2009 were selected from the Netherlands Cancer Registry (NCR) and included in this study. The NCR is a database on cancer diagnosis and treatment hosted by the Netherlands Comprehensive Cancer Organization (IKNL). It receives reports of diagnosed malignancies from the nationwide network and registry of histopathology and cytopathology in the Netherlands (PALGA), which are confirmed and completed by the national hospital discharge databank. The interval 2003–2009 was chosen to allow sufficiently long follow‐up.

Trained data managers collect data on diagnosis, staging and treatment from medical records using international coding rules. Breast cancer stage is defined according to the sixth edition of the TNM classification of malignant tumours^20^. Clinical tumour or node category was used when pathological stage was unknown. Oestrogen receptor and progesterone receptor status was considered positive if at least 10 per cent positive nuclear staining of tumour cells was demonstrated. Information on co‐morbidity was collected for this study, but only for patients diagnosed in 2007–2009 for logistic reasons. For patients diagnosed between 2003 and 2006, data on co‐morbidity were available only for those diagnosed in one of the nine regions in the Netherlands, as this is the only region that regularly collects such information. Missing co‐morbidity data for the other regions were imputed (see below). Vital status was available until 31 January 2017 through linkage of NCR data with the Municipal Personal Records database.

### Hospital variation

In clinical practice, the decision to omit surgery is based on disease characteristics, age, co‐morbidity, and other aspects of general health and frailty, such as physical, cognitive and social functioning. As these latter factors are generally not measured or well recorded in observational databases, statistical techniques such as multivariable analysis or propensity score matching cannot fully adjust for them, leaving residual confounding. Previous studies^21^, ^22^, ^23^ have demonstrated that residual confounding can lead to implausible results. To minimize confounding, an instrumental variable approach was used. Under certain assumptions, this method can adjust for unmeasured confounding. Variation in the percentage of patients undergoing primary surgery across hospitals (the instrument) was used, and outcomes of patients treated in hospitals with different rates of primary surgery were compared^22^. Hospital was used as instrument as rates of primary surgery varied substantially across hospitals, and no major differences in case mix between hospitals were expected as all hospitals in the Netherlands provide breast cancer care and older patients are assumed to go to the hospital nearest their home. Therefore, groups of hospitals are similar with respect to patients' prognosis and general health, and potential differences in outcomes can be attributed to the difference in surgery rates. Hospitals that contributed fewer than ten patients were excluded.

Three groups were defined by dividing 117 hospitals based on rates of primary surgery while ensuring equal numbers of patients in each group: hospitals with higher rates (range 75·9–100 per cent), moderate rates (63·2–75·8 per cent) and lower rates (37·6–63·1 per cent). Those treated in these hospitals are referred to as patients treated in hospitals with higher, moderate and lower rates of surgery respectively. The rate of surgery is defined as the rate of primary surgery. To evaluate the effect of using hospital variation to minimize confounding, patient characteristics of the three groups were compared.

### Statistical analysis

Multiple imputation by chained equation was performed to account for missing values of grade, human epidermal growth factor receptor (HER) 2 status and co‐morbidity. Missing values for these variables were assumed to be missing at random after examination of patterns^24^. Imputation models were applied including all variables as predictors. Results were based on the pooled results of 25 imputed sets according to Rubin's rules^25^. Pearson's χ^2^ tests were used to assess differences in patient characteristics between groups.

In observational data, the time between diagnosis and the start of treatment is ‘immortal time’ as a patient had to survive this period to start the treatment. As the time to treatment was immortal for patients who underwent surgery in this study, a landmark approach was used to avoid immortal time bias^26^, ^27^. Hence, follow‐up time started 60 days after diagnosis. Patients who died before this landmark were excluded from the survival analysis. Follow‐up ended at the date of death or last follow‐up visit.

As older patients with breast cancer often die from causes other than those related to breast cancer, the primary outcome was relative survival. Relative survival was used as proxy for breast cancer‐specific survival (BCSS) as cause of death is not available in the NCR. Moreover, ascertaining cause of death in older patients is susceptible to misclassification bias^28^. Relative survival is calculated by dividing the observed survival in a patient population by the expected survival in the general population matched by age, sex and year of diagnosis^29^. Hence, relative survival takes into account the patient population's background mortality and in the present study expresses the excess risk of death owing to breast cancer. Relative survival estimates cancer‐specific survival under the condition that the general population's mortality is representative of the background mortality in the patient population. In other words, the prevalence of co‐morbid diseases should be similar in the patient population and the general population. Relative survival is considered a reliable outcome in older patients with breast cancer as it has been demonstrated that the prevalence of co‐morbid diseases is indeed comparable among patients with breast cancer and those without cancer^30^. To compare relative survival, relative excess risks with 95 per cent confidence intervals were calculated using generalized linear Poisson models. Patients treated in hospitals with higher rates of surgery were used as reference group.

Kaplan–Meier estimates of overall survival were calculated. To compare overall survival, hazard ratios (HRs) with 95 per cent confidence intervals were calculated using Cox proportional hazard models. Patients treated in hospitals with higher rates of surgery were used as reference group. In addition, to explore different effects of omission of surgery in patients with and without co‐morbidity, a stratified analysis was performed in groups with a Charlson Co‐morbidity Index (CCI) score^31^ of 0 or at least 1. As a statistically significant age difference across the groups remained despite applying the instrumental variable approach to reduce confounding, a multivariable analysis including age was undertaken. The proportionality assumption was tested by plotting the scaled Schoenfeld residuals. No violation of the assumption was found.

All statistical tests were two‐sided and *P* < 0·050 was considered statistically significant. Statistical analysis was done with SPSS® version 23.0 (IBM, Armonk, New York, USA) and Stata® version 12.1 (StataCorp, College Station, Texas, USA).

## Results

A total of 6464 older patients with stage I–II hormone receptor‐positive breast cancer were included. Overall, 4465 patients (69·1 per cent) underwent surgery and 1999 (30·9 per cent) did not. There were differences in characteristics between the two groups (*Table* 1). Patients who did not have surgery were more often older; 69·2 per cent of these patients were aged 85 years or older compared with 35·7 per cent of patients who had surgery (*P* < 0·001). Among patients who did not undergo surgery, 58·3 per cent had a CCI score of 1 or more, compared to 45·7 per cent of those who had surgery (*P* < 0·001). No differences in stage, grade or HER2 status were observed after multiple imputation (*Table* 1). Of the patients who did not have surgery, 94·1 per cent received primary endocrine treatment.

**Table 1:** Characteristics of patients who were treated with or without primary surgery

	Surgery (*n* = 4465)	No surgery (*n* = 1999)	P*
**Age (years)**			< 0·001
80–84	2870 (64·3)	615 (30·8)	
85–89	1324 (29·7)	829 (41·5)	
≥ 90	271 (6·1)	555 (27·8)	
**CCI score**			< 0·001
0	980 (21·9; 54·3)	510 (25·5; 41·7)	
1	468 (10·5; 26·7)	386 (19·3; 31·6)	
≥ 2	323 (7·2; 19·0)	321 (16·1; 26·8)	
Unknown	2694 (60·3)	782 (39·1)	
**TNM stage**			0·866
I	1458 (32·7)	657 (32·9)	
II	3007 (67·4)	1342 (67·1)	
**Tumour grade**			0·159
1	1098 (24·6; 26·2)	101 (5·1; 31·5)	
2	2306 (51·6; 55·2)	180 (9·0; 51·8)	
3	784 (17·6; 18·6)	61 (3·1; 16·7)	
Unknown	277 (6·2)	1657 (82·9)	
**HER2 status**			0·689
Positive	217 (4·9; 7·4)	79 (4·0; 7·8)	
Negative	2864 (64·1; 92·6)	977 (48·9; 92·2)	
Unknown	1384 (31·0)	943 (47·2)	

Values in parentheses are percentages including missing data; percentages after multiple imputation. CCI, Charlson Co‐morbidity Index; HER2, human epidermal growth factor receptor 2. *Pearson's χ^2^ test.

Rates of surgery were on average 82·6, 69·7 and 54·8 per cent in the hospitals with higher, moderate and lower rates of surgery respectively. Furthermore, 15·2, 28·5 and 43·6 per cent received primary endocrine treatment, whereas 2·1, 1·8 and 1·6 per cent received no treatment (*Table* 2; *Fig*. *S1*, supporting information). Patients treated in hospitals with lower rates of surgery were more often older than patients treated in hospitals with moderate and higher rates (48·5 per cent aged 85 years or more *versus* 46·1 and 43·7 per cent respectively; *P* = 0·003). No other differences were observed across the groups.

**Table 2:** Characteristics of patients who were treated at hospitals with higher, moderate or lower rates of primary surgery

	Higher rates (*n* = 2159)	Moderate rates (*n* = 2158)	Lower rates (*n* = 2147)	P*
**Treatment**				
Surgery	1784 (82·6)	1505 (69·7)	1176 (54·8)	
Primary endocrine treatment	329 (15·2)	615 (28·5)	937 (43·6)	
No treatment	46 (2·1)	38 (1·8)	34 (1·6)	
**Age (years)**				0·003
80–84	1216 (56·3)	1163 (53·9)	1106 (51·5)	
85–89	705 (32·7)	722 (33·5)	726 (33·8)	
≥ 90	238 (11·0)	273 (12·7)	315 (14·7)	
**CCI score**				0·985
0	448 (20·8; 50·5)	488 (22·6; 50·2)	554 (25·8; 50·6)	
1	260 (12·0; 27·9)	293 (13·6; 29·0)	301 (14·0; 27·7)	
≥ 2	209 (9·7; 21·6)	198 (9·2; 20·8)	237 (11·0; 21·8)	
Unknown	1242 (57·5)	1179 (54·6)	1055 (49·1)	
**TNM stage**				0·215
I	680 (31·5)	705 (32·7)	730 (34·0)	
II	1479 (68·5)	1453 (67·3)	1417 (66·0)	
**Tumour grade**				0·511
1	475 (22·0; 28·1)	389 (18·0; 27·2)	335 (15·6; 28·3)	
2	946 (43·8; 54·1)	878 (40·7; 55·9)	662 (30·8; 52·4)	
3	318 (14·7; 17·8)	257 (11·9; 16·8)	270 (12·6; 19·3)	
Unknown	420 (19·5)	634 (29·4)	880 (41·0)	
**HER2 status**				0·554
Positive	96 (4·4; 7·7)	104 (4·8; 7·8)	96 (4·5; 7·1)	
Negative	1252 (58·0; 92·3)	1246 (57·7; 92·2)	1343 (62·6; 92·9)	
Unknown	811 (37·6)	808 (37·4)	708 (33·0)	
**RT after BCS**				0·066
Yes	251 (70·3)	310 (71·1)	234 (77·7)	
No	106 (29·7)	126 (28·9)	67 (22·3)	
**RT after mastectomy**				0·298
Yes	67 (4·7)	64 (6·0)	51 (5·8)	
No	1360 (95·3)	1005 (94·0)	824 (94·2)	
**Adjuvant endocrine therapy**				0·627
Yes	1015 (56·9)	875 (58·1)	663 (56·4)	
No	769 (43·1)	630 (41·9)	513 (43·6)	
**Adjuvant chemotherapy**				–
Yes	7 (0·3)	1 (< 0·1)	1 (< 0·1)	
No	2152 (99·7)	2157 (> 99·9)	2146 (> 99·9)	

Values in parentheses are percentages including missing data; percentages after multiple imputation. CCI, Charlson Co‐morbidity Index; HER2, human epidermal growth factor receptor 2; RT, radiotherapy; BCS, breast‐conserving surgery. *Pearson's χ^2^ test.

**Figure 1:**
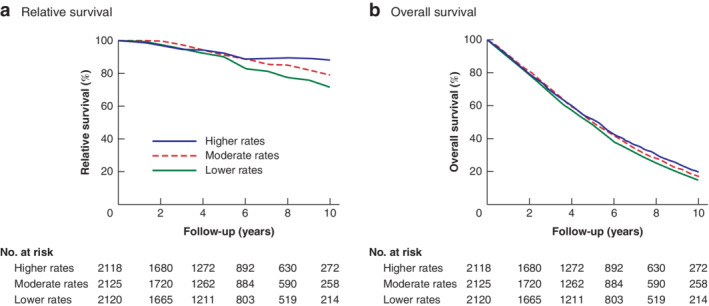
Cumulative relative survival and overall survival of patients treated in hospitals with different rates of primary surgery **a** Relative and **b** overall survival.

Of the 6464 patients, 6363 were included in the survival analysis as six patients were lost to follow‐up and 95 died in the first 60 days after diagnosis. Relative survival is shown in *Fig*. 1 *a*. Relative survival was lower for patients treated in hospitals with lower compared with higher rates of surgery (90·2 *versus* 92·4 per cent after 5 years; 71·6 *versus* 88·2 per cent after 10 years) (*Table* 3). Compared with the reference group of patients treated in hospitals with higher rates of surgery, the relative excess risk of death was 2·00 (95 per cent c.i. 1·17 to 3·40) for patients treated at hospitals with lower rates (*Table* 3). Of note, the relative survival curves are overlapping for the first 5 years (*Fig*. 1 *a*).

**Table 3:** Relative survival and relative excess risk for patients treated in hospitals with different rates of primary surgery

		Relative survival (%)			
	Surgically treated patients (%)	5 years	10 years	Relative excess risk*	*P*
					0·019
Higher rates	82·6	92·4 (88·5, 96·2)	88·2 (80·4, 96·3)	1·00 (reference)	
Moderate rates	69·7	91·1 (87·2, 95·0)	79·0 (71·4, 86·8)	1·29 (0·70, 2·39)	
Lower rates	54·8	90·2 (86·2, 94·2)	71·6 (64·1, 79·4)	2·00 (1·17, 3·40)	

Values in parentheses are 95 per cent confidence intervals. *Model included all available follow‐up.

Overall survival rates were also lower for patients treated in hospitals with lower compared with higher rates of surgery (48·3 *versus* 51·3 per cent after 5 years; 15·0 * versus* 19·8 per cent after 10 years) (*Fig*. 1 *b* and *Table* 4). Compared with the reference group of patients treated in hospitals with higher rates of surgery, the adjusted HR for death was 1·07 (95 per cent c.i. 1·00 to 1·14) for patients treated at hospitals with lower rates (*Table* 4). Stratified by co‐morbidity, the adjusted HR for death among patients treated in hospitals with lower compared with higher rates of surgery was 1·05 (0·95 to 1·16) in patients with a CCI score of 0, and 1·08 (0·98‐1·20) among those with a CCI score of at least 1.

**Table 4:** Cox proportional hazards analysis for overall survival of patients treated in hospitals with different rates of primary surgery stratified by co‐morbidity

		Overall survival (%)					
	Surgically treated patients (%)	5 years	10 years	Hazard ratio*	*P*	Age‐adjusted hazard ratio*	*P*
**All patients**					0·003		0·135
Higher rates	82·6	51·3 (49·2, 53·4)	19·8 (18·0, 21·6)	1·00 (reference)		1·00 (reference)	
Moderate rates	69·7	49·9 (47·8, 52·0)	17·2 (15·5, 18·9)	1·04 (0·98, 1·12)		1·03 (0·96, 1·09)	
Lower rates	54·8	48·3 (46·2, 50·4)	15·0 (13·4, 16·7)	1·12 (1·05, 1·20)		1·07 (1·00, 1·14)	
**CCI score 0**					0·060		0·646
Higher rates	88·2	60·4 (59·8, 61·0)	25·9 (25·3, 26·4)	1·00 (reference)		1·00 (reference)	
Moderate rates	74·4	57·9 (57·3, 58·5)	22·4 (21·9, 22·9)	1·06 (0·96, 1·18)		1·02 (0·91, 1·13)	
Lower rates	60·2	56·5 (55·9, 57·1)	20·1 (19·6, 20·6)	1·13 (1·03, 1·25)		1·05 (0·95, 1·16)	
**CCI score ≥ 1**					0·143		0·323
Higher rates	76·7	40·1 (39·7, 40·5)	11·5 (11·2, 11·8)	1·00 (reference)		1·00 (reference)	
Moderate rates	64·7	40·7 (40·2, 41·1)	11·2 (10·9, 11·5)	1·02 (0·99, 1·14)		1·02 (0·92, 1·15)	
Lower rates	48·8	39·1 (38·7, 39·5)	10·3 (10·0, 10·6)	1·10 (1·00, 1·22)		1·08 (0·98, 1·20)	

Values in parentheses are 95 per cent confidence intervals. *Model included all available follow‐up. CCI, Charlson Co‐morbidity Index.

## Discussion

This study showed that omission of surgery had no effect during the first 5 years of follow‐up, but was associated with worse relative and overall survival after 5 years in patients aged 80 years or older with stage I–II hormone receptor‐positive breast cancer.

These findings support the recommendation of international guidelines that primary endocrine treatment is an alternative for patients with a life expectancy of 2–3 years, although, based on the data presented here, it could be argued that primary endocrine treatment is justified in patients with a life expectancy up of to 5 years. In a systematic review^12^ of six RCTs comparing surgery and tamoxifen monotherapy, only one trial^13^ demonstrated a survival advantage in favour of surgery. Findings of the present study are in line with results from that trial, although with the finding of similar survival during the first 3 years compared with 5 years in the present study. The emergence of aromatase inhibitors might have improved the efficacy of primary endocrine treatment and contributed to this difference. This is substantiated by the findings of a cohort study^18^ in which 616 patients received primary endocrine treatment during the years when aromatase inhibitors were introduced; although 69·3 per cent of the patients received tamoxifen as first‐line agent, the study demonstrated a median time to progression of 49 (range 4–132) months^18^. It is important to recognize that the early trials included only patients aged 70 years or more who were considered fit for surgery, whereas all patients aged 80 years or older in the Netherlands, including frail patients, were included in the present population‐based cohort study. Because of this, the burden of mortality from non‐breast cancer‐related causes was considerably higher here, which could explain why the effect on survival was seen after a longer period.

There are no randomized data available comparing surgery and aromatase inhibitor monotherapy. The ESTEem (Endocrine +/– Surgical Therapy for Elderly women with Mammary cancer) trial was initiated to compare anastrozole with and without surgery, but unfortunately had to close owing to poor accrual. Patient preference for a specific treatment may have contributed to the disappointing accrual. Furthermore, in clinical practice, omission of surgery is generally considered in frail older patients and the participation of this patient group in RCTs is often poor.

Several observational studies have compared outcomes of patients treated with primary surgery or primary endocrine treatment. The majority demonstrated superior BCSS and overall survival in patients who had primary surgery^32^, ^33^. Only one study^18^ did not report a difference in 5‐year BCSS between patients who had surgery *versus* primary endocrine treatment among those aged 80 years or more. Residual confounding owing to differences in general health and frailty between patients who had primary surgery and those who received primary endocrine treatment is usually not measured in observational databases, which makes direct comparisons at risk of bias.

In the present study, patients treated with and without primary surgery were not compared directly; instead, outcomes were compared in groups of patients treated in hospitals with different rates of primary surgery. As the measured patient and tumour characteristics were similar across the groups, the amount of residual confounding by unmeasured factors was reduced. An instrumental variable approach, however, requires further assumptions, such as similar quality of hospital care^34^. With a difference of 27·8 per cent in omission of surgery between the hospitals with higher and lower rates of surgery, both relative survival and overall survival were worse for patients treated in the hospitals with lower rates. As expected, overall survival rates are lower than relative survival rates owing to the high population mortality in this age group. Consequently, the impact of omission of surgery on relative survival translates into a smaller impact on overall survival, and for some patients with high competing mortality risks this absolute benefit is likely small enough to justify omission of surgery. On the other hand, the present data suggest that, if rates of surgery in patients aged 80 years and older were to increase, survival after 5 years may improve.

Given the overlapping survival curves, the present data may suggest that omission of surgery can be considered in patients with a life expectancy below 5 years. Yet, even in patients with limited life expectancy, there are reasons for being reluctant to offer primary endocrine treatment as an alternative to surgery. Endocrine therapy often has side‐effects, such as hot flushes, joint pain and fatigue, which can impair activities of daily living and quality of life^10^, ^11^. Furthermore, in the adjuvant setting, non‐persistence with endocrine therapy has been demonstrated to increase with older age^35^. As patients with favourable tumour characteristics (grade 1 up to 2 cm in size; grade 2 up to 1 cm) do not receive adjuvant endocrine treatment in the Netherlands, such patients can be spared endocrine therapy completely after primary surgery.

Another disadvantage of primary endocrine treatment is that it is only effective for a limited period, after which a switch of treatment is needed. Although different lines of endocrine treatment are available, surgery may eventually be necessary. Furthermore, whereas primary endocrine treatment requires long‐term regular hospital visits to evaluate disease progression, few hospital visits are required after surgery. The main advantage of primary endocrine treatment over surgery is that the risks and inconvenience of surgery can be avoided. Breast surgery, however, is associated with low morbidity rates, and age itself is not a risk factor for postoperative complications^36^, ^37^. The inconvenience of primary endocrine treatment may persist for a long time, whereas the inconvenience of having surgery is generally temporary. Accurately estimating life expectancy is not straightforward. In 2018, the life expectancy of a Dutch woman aged 70 years was 17·3 years, and for a woman aged 80 years was 9·9 years^19^. Certain co‐morbidities can decrease life expectancy, but impaired cognition, malnutrition and dependency in activities of daily living are also important predictors^38^. As these factors may not always be recognized, a geriatric assessment is advisable^39^. The present findings underline that estimating life expectancy is important for optimal treatment decisions, but unfortunately this is often difficult for patients aged over 80 years.

Strengths of this study were that hospital variation was used to minimize confounding by indication as much as possible, and relative survival was calculated, which takes into account mortality from other causes. All consecutive patients in a large, nationwide cohort were included with detailed information on tumour characteristics and co‐morbidity. Limitations of this study were related to the data and methodology. Information on treatments was limited to the first year after diagnosis, and it is therefore unknown how many patients eventually had surgery after primary endocrine treatment. No information on specific endocrine agents and successive lines of endocrine therapy was available. Inherent to following the instrumental variable approach using hospital variation in rates of primary surgery, only the impact of a difference in rate of surgery of 27·8 per cent could be assessed, which reduced the statistical power. Although this was sufficient to demonstrate a survival difference in the primary analysis, the findings for the stratified analysis suggest a lack of power. Although confounding by unmeasured factors can theoretically be avoided using the instrumental variable approach, an instrument that meets all of the required assumptions is not always available in clinical data^23^, ^34^. There was a small age difference across the groups in the present study. Although age was adjusted for in multivariable analysis, residual confounding could not be ruled out completely^34^. Future research is needed to evaluate the side‐effects of primary endocrine treatment using aromatase inhibitors, compliance and treatment switches, and to compare quality of life and physical functioning of patients treated with surgery or primary endocrine therapy.

## Supporting information

Fig. S1 Primary treatment in hospitals with higher, moderate and lower surgery rates.
