# Supplementary material for: Effect of omission of surgery on survival in patients aged 80 years and older with early‐stage hormone receptor‐positive breast cancer
Source: Br J Surg. 2020 Apr 7;107(9):1145–53. doi: 10.1002/bjs.11568 (PMC7496090; doi:10.1002/bjs.11568)
Supplement: Supplementary file 1 — Fig. S1 Primary treatment in hospitals with higher, moderate and lower surgery rates. [file BJS-107-1145-s001.docx]

**BJS11568**

**Effect of omission of surgery on survival in patients aged 80 years and older with early-stage hormone receptor-positive breast cancer**

A. Z. de Boer, N. A. de Glas, P. J. Marang-van de Mheen, O. M. Dekkers, S. Siesling, L. de Munck, K. M. de Ligt, G. J. Liefers, J. E. A. Portielje and E. Bastiaannet

**Fig. S1 Primary treatment in hospitals with higher, moderate and lower surgery rates.**


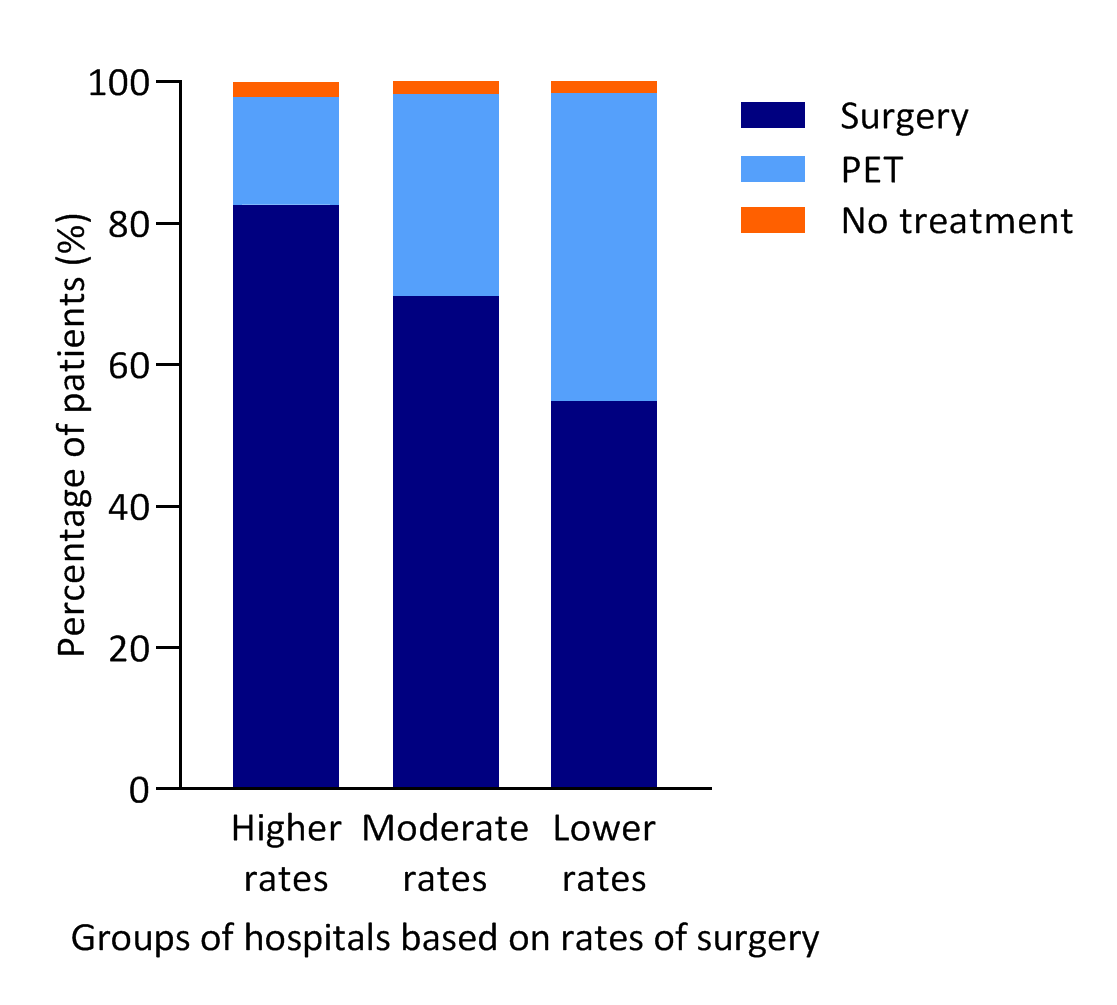


**PET, primary endocrine therapy.**
